# Context Regulation of Mind Wandering in ADHD

**DOI:** 10.1177/1087054720956714

**Published:** 2020-09-18

**Authors:** Natali Bozhilova, Giorgia Michelini, Christopher Jones, Jonna Kuntsi, Katya Rubia, Philip Asherson

**Affiliations:** 1 King’s College London, De Crespigny Park, UK; 2 University of California Los Angeles, USA

**Keywords:** ADHD, mind wandering, working memory, sustained attention, context regulation

## Abstract

**Objective:**

We aimed to understand the association between MW frequency and clinical measures, context regulation of MW and group differences in task performance.

**Method:**

27 adults with ADHD and 29 controls performed tasks manipulating demand on working memory and sustained attention, and recorded their MW frequency using probes.

**Results:**

A significant association between MW frequency and the clinical measures was demonstrated. Along with increased MW frequency, individuals with ADHD reported decreasing MW frequency during increasing demands on working memory (context regulation), but not on sustained attention (deficient context regulation). Controls, however, maintained continuous task focus across all conditions. Group differences in task performance were no longer significant after adding MW frequency as a covariate.

**Conclusion:**

Deficient context regulation during increasing demands on sustained attention suggests that sustained attention deficits may play a more important role in regulation of MW in ADHD. MW frequency might also underpin performance deficits in ADHD.

## Introduction

ADHD is a common neurodevelopmental disorder affecting 5% to 7% of children (Sayal et al., 2018; Polanczyk et al., 2014). Prevalence estimates for ADHD in adults range from 2.5% to 3.4% (Fayyad et al., 2017; Simon et al., 2009) with the most recent review reporting an average prevalence of 2.8% for DSM-4ADHD (Fayyad et al., 2017). Diagnostic criteria for ADHD focus on impairing levels of inattentive and hyperactive-impulsive behaviors. These criteria reflect the behavioral symptoms commonly used to describe children with ADHD, but do not fully capture the experience of adults. Clinical observations of adults with ADHD describe poorly controlled and excessive mind wandering (MW) (Asherson, 2005), which strongly predicts spontaneous before but not deliberate MW (Mowlem et al., 2019; Seli et al., 2015). Experimental experience-sampling (Franklin et al., 2017; Seli et al., 2015; van den Driessche et al., 2017) and self-report measures of MW (Biedermann et al., 2019; Mowlem et al., 2016, 2019) have also demonstrated increased frequency of spontaneous MW (MW-S) in individuals with ADHD compared to controls. MW-S reflects unintentional inattention during a task, which is detrimental to task performance (Franklin et al., 2017; Seli et al., 2015), suggesting that MW underlies core attentional processes in ADHD.

An important aspect of MW is context regulation, which occurs when MW frequency decreases as task demands increase, in order to allow for an optimal task performance (Smallwood & Andrews-Hanna, 2013). Context regulation was first demonstrated using population and college samples, which showed greater MW frequency during the 0-back condition (no working memory load) compared to the 1-back condition in (working memory load) of an attention task (Konishi et al., 2015; Smallwood et al., 2013; Thomson et al., 2013). A further study found that MW was more frequent under very low and very high cognitive demand conditions, compared to moderate cognitive demand (Randall et al., 2019).

Another important aspect of context regulation of MW is its relationship with executive control (e.g., working memory capacity). One proposal is that excessive MW results from a failure in executive control to prevent automatic MW from becoming conscious (McVay & Kane, 2012). In line with this model, lower MW frequency under high cognitive demand conditions was associated with increased working memory capacity (Kam & Handy, 2014; Kane, Brown, et al., 2007; Kane, Conway, et al., 2007) and fewer incorrect responses during a high demand 3-back working memory task (Rummel & Boywitt, 2014). An alternative hypothesis is that good executive control skills (e.g., working memory capacity) maintains personally salient task-unrelated thoughts during low cognitive demand conditions, and supports a decrease in MW frequency during high demand conditions (Smallwood, 2010; Smallwood & Schooler, 2006). These findings suggest that varying or/and working memory capacity modulates the frequency of MW. However, no previous study has investigated the context regulation of MW in individuals with ADHD.

Neuroimaging and electrophysiological evidence suggest that individuals with ADHD experience deficient context regulation of neural activity (Bollmann et al., 2017; Christakou et al., 2013; Michelini et al., 2019; Skirrow et al., 2015). In particular, compared to controls, individuals with ADHD failed to show an increase in theta power (Rommel et al., 2016; Skirrow et al., 2015) and decreased activity in areas of the default mode network (DMN) (Christakou et al., 2013) with increasing demands on tasks of sustained attention and working memory (Bollmann et al., 2017). We therefore proposed that deficient context regulation of neural activity may underlie poor context regulation of MW in ADHD (i.e., increased MW frequency irrespective of increasing task demands) (Bozhilova et al., 2018). However, these studies did not measure MW, with an experimental experience sampling approach using thought probes that enquire about whether the individual is mind wandering or focused on the task.

To address this question, we studied adults with and without ADHD adopting an experience sampling approach during two cognitive tasks: the Mind Wandering Task (MWT) and Sustained Attention Task (SAT). The MWT was previously used to demonstrate context regulation of MW in population-based samples (Konishi et al., 2015), whereas the SAT has previously shown that context regulation of neural activity is deficient in individuals with ADHD (Christakou et al., 2013).

Our first aim was to test the association between the experimental experience sampling measures of MW with clinical measures of MW, ADHD, executive skills, and functional impairment (Analysis 1). Our second was to study frequency of MW during changing task demands and context regulation of MW in individuals with ADHD compared to controls (Analysis 2). Our third aim was to compare cognitive performance between groups and test whether MW would explain statistically any between-group differences (Analysis 3).

## Method

### Participants

The sample consisted of 56 individuals (27 with ADHD and 29 controls) of mixed gender and between the ages of 18 and 65 years. The groups were matched on age, sex, and IQ (Table 1).

**Table 1.** Comparison Between ADHD and Control Groups on Demographic Characteristics.

	ADHD	Controls	*d*	*p*
	Mean ± *SD*	Mean ± *SD*		
Age (years)	37 ± 8.67	32 ± 11.42	0.49	.06
IQ	111.11 ± 12.43	113.66 ± 16.08	0.14	.51
	Males:Females	Males:Females	Chi^2^	*p*
Gender	16:11	14:15	0.68	.29

*Note.* IQ = Intelligent Quotient from the Wechsler Abbreviated Scale of Intelligence, WASI-II.

The adults with ADHD were recruited from the South London and Maudsley NHS Trust ADHD clinic, the Barnet, Enfield and Haringey Mental Health Trust clinics, online advertisements via adult ADHD networks and primary care physicians. Control adults without ADHD and no prior diagnosis or treatment for any mental health condition were recruited via online recruitment advertisements from all over London. Participants in both groups were excluded if they had a current or past diagnosis of major physical illness (e.g., neurological problems, head injury), severe recurrent mental health problems other than ADHD (e.g., psychosis, schizophrenia, bipolar disorder, antisocial personality disorder), current or past substance abuse (defined as more than 8 units for males or 6 units for females of alcohol consumed daily, or recreational drug use more than twice weekly), or an IQ < 80.

All ADHD participants had a formal diagnosis of ADHD based on clinical records and met both DSM-4 and DSM-5 criteria for ADHD, confirmed during assessments for this study. Fourteen participants with ADHD were receiving pharmacological treatment for ADHD. Twelve were receiving stable treatment with stimulant medication and two with atomoxetine. Seven participants with ADHD experienced comorbid difficulties with depression and anxiety and were taking a low dose of a concomitant medication for anxiety or depression (SSRIs). Two individuals with ADHD also had a suspected autism spectrum disorder. All these nine individuals were included in the final sample.

### Procedure

All participants were invited for a test session lasting approximately 3 to 4 hr, which involved a diagnostic interview for ADHD (Supplemental Material 1), a cognitive task battery comprising two tasks (1 hr 30 min in total including breaks and a training block for each task; with simultaneous EEG recordings not used in the current study), IQ testing (vocabulary and matrix reasoning from the Wechsler Abbreviated Scale of Intelligence—II [WASI-II]) and self-report questionnaires (Supplemental Material 2). Participants on ADHD medication (both stimulants and non-stimulants) were asked to refrain from taking their ADHD medication for 48 hr before the assessment. All participants were asked to refrain from consuming caffeine, alcohol, illicit and non-illicit substances or smoking on the day of assessments and the preceding evening.

### Cognitive Tasks

#### Mind wandering task

**Figure 1.**
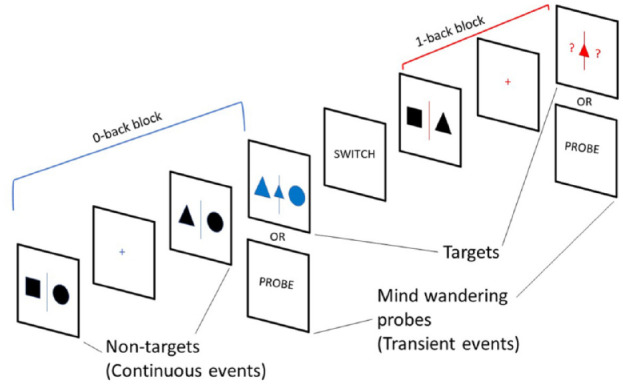
Schematic representation of the Mind Wandering Task (MWT). Participants alternated between the two conditions. One condition involved observing two black shapes (non-target) before three blue shapes (target) appeared. At that point, the participant had to indicate which of the two side shapes matches the small blue shape in the middle (choice reaction, 0-back). In the 1-back condition, participants had to encode in working memory the two black shapes and when a small red shape with two red question marks on each side appears, they had to choose the left or right question mark based on the position of the black shape that is identical to the small red shape in the prior trial (working memory, 1-back) (Konishi et al., 2015).

The 0-back (choice reaction) condition measures general alertness and motor speed, whereas the 1-back condition measures visual working memory performance (Konishi et al., 2015) (Figure 1). In the 0-back condition, participants observed a sequence of black shapes (separated with a blue line into a right and a left shape) in the middle of the computer screen while waiting for a blue target (a small shape with two bigger shapes on each side). Upon target presentation, they had to indicate the location of the bigger shape which matched the small target shape by pressing the left or the right arrow. In the 1-back condition, participants were exposed to the same sequence of black shapes (separated by a red line into a right and a left shape) and were intermittently presented with two red question marks (“?”) with a small red shape (target) between the question marks. When the question marks appeared, the participants had to make a manual response to indicate the location (left or right) of the shape in the previous trial that was identical to the small target shape. Because the occurrence of the colored question marks was randomly determined, this task required participants to encode and retain in memory the location (left or right) of each non-colored shape (Figure 1).

The order of conditions was counterbalanced. For each trial, between 2 and 6 non-targets preceded the target. The non-targets lasted for 1 to 3 s with increasing steps of 0.1 s in each trial (the maximum interval length was 3 s for each trial). The total number of stimuli was 128 targets (64 in each condition) and 580 non-targets (290 in each condition). Each target lasted for 4 s, allowing the participant 4 s to respond until their response ended it immediately. The fixation appeared before and after all task stimuli crosses ranged from 2 to 4 s with increasing steps of 0.1 s.

There was a total of eight trials in each block for each condition. There were eight blocks, with a varying duration from 40 to 120 s. At the end of each block, participants were informed that they were about to start a new block with either the same condition with the word “STAY” or that they were about to switch to the other condition with the word “SWITCH.” Both message words “SWITCH” and “STAY” appeared on the screen for 5 s. The total duration of task was approximately 30 min divided into two 15-min sessions.

#### Sustained Attention task

**Figure 2.**
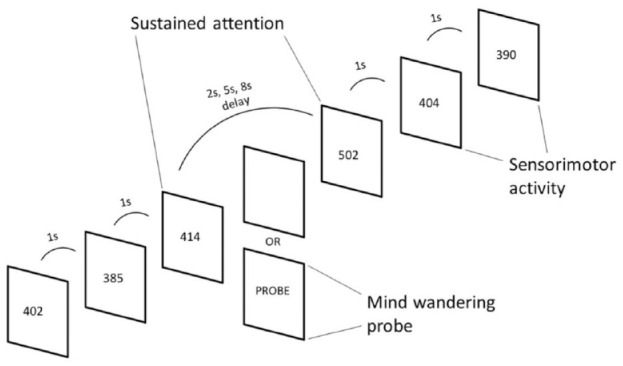
Schematic representation of the Vigilance/Sustained Attention Task (SAT). Individuals were asked to respond as fast as possible to the appearance of black-counters (participant’s reaction time) on the screen that count up in milliseconds. The counters appeared either after frequent and predictable delays of 1 s in blocks of 3 to 5 stimuli, or after unpredictable long delays of 2, 5, or 8 s, pseudorandomly interspersed into the blocks of 1 s delays (Christakou et al., 2013).

The SAT is a vigilance task, which has three levels of a progressively increasing sustained attention load (2, 5, and 8 s). The participants are required to respond as quickly as possible to the appearance of a counter (i.e., black digits) of milliseconds, via a right button response within 1 s. The visual stimuli appeared either after short, frequent consecutive intervals of 1 s, in series of 3 to 5 stimuli (520 in total, 260 in each session), or after longer, less frequent time delays of 2, 5, or 8 s (52 in total, 26 each in each session), pseudo-randomly interspersed into the blocks of 3 to 5 trials of 1 s (Christakou et al., 2013 ) (Figure 2). The long, infrequent delays place a higher load on sustained attention/vigilance, whereas the short, frequent 1-s delays are typically anticipated and place higher demand on sensorimotor synchronization (Christakou et al., 2013). The total duration of the task was approximately 30 min divided into two 15-min sessions.

The SAT design and approach contrasts other attentional task (e.g., Sustained Attention to Response Task (SART) (O’Connell et al., 2009); Fast Task (Kuntsi et al., 2005)) that have predictable, same-length intervals. Such tasks have elicited greater MW frequency in population-based samples with time-on-task (Randall et al., 2014; Thomson et al., 2015) and slower and more variable responses in individuals with ADHD (Andreou et al., 2007; Michelini, Cheung, et al., 2018). In contrast, due to the unpredictability and variety of the inter-stimulus delays in the SAT, the delays are expected to elicit increased task focus in controls and enhanced MW frequency in ADHD with increasing delays.

#### MW probes

MW was recorded using thought probes (15 per session, 30 in total) at approximately 1-min intervals. The probe appeared in the place of the targets in the MWT and in the place of the stimulus following the infrequent delays in the SAT. We included 26 delays per session (78 in total) contrasting 20 delays (60 in total) in the original version of the SAT. Most of these extra delays (36 in total) were followed by thought probes (30 in total) rather than the task stimulus (black digits), ensuring consistency in the number of delays between our and the original version of the SAT. Participants were first asked “*Where was your attention just before this probe?*” with two response options “*On task*” and “*Off task.*” If they had responded “Off task,” another question enquired “*Were you aware of your attention drifting away from the task?*” with two responses options “*Aware*” and “*Unaware.*” The use of thought probes to measure MW has been validated in previous neuroimaging studies contrasting changes in neural function between periods of task-focus and off task thoughts (Kirschner et al., 2012; Smallwood, Beach et al., 2008; Smallwood, McSpadden, et al., 2008).

MW frequency was calculated as a proportion using the number probes indicating MW divided by the total number of probes. The values ranged from 0 to 1, equivalent to 0% to 100% of the time.

#### Task performance

For each task and condition, cognitive performance was measured using mean reaction time (MRT), intra-subject reaction time variability (RTV), and error rate. For the MWT, we measured accuracy/errors (total number of incorrectly chosen shape to match the target), based on previous work (Konishi et al., 2015) reporting only this kind of errors for this task. Working memory capacity was quantified as the difference in incorrect responses/accuracy between the 1-back and 0-back conditions in the MWT (i.e., 1-back errors − 0-back errors), based on previous literature using the same measure (Dodds et al., 2011; Hur et al., 2017).

For the SAT, participants had only one response option during, before and after the appearance of task stimulus. We therefore measured the proportion of non-responses (i.e., omission errors) out of the number of trials for each delay type separately, as an index of sustained attention. After the end of each delay/interval, there was a stimulus and a response (a delay-affected trial). We calculated all SAT measures based on this trial (a stimulus and a response following straight after the duration of the delay) to study the effect of delay type. Unlike in a previous study of children with ADHD (Christakou et al., 2013), premature responses were rare in our adult sample (less than 5 per participant) and were therefore not examined in this study.

### Statistical Analyses

MRT and RTV variables showed a normal distribution. Error data (incorrect responses/accuracy and omission errors in the MWT and SAT, respectively) were positively skewed and transformed using a log transformation. In order to report standardized beta coefficients, all variables were also standardized before analyses.

Analysis 1: To test the relationship between the experimental experience sampling of MW and the clinical measures, we carried out linear regressions using total MW frequency during each task as an independent variable and ADHD symptoms (total number of inattentive and hyperactive-impulsive symptoms as reported in the DIVA), self-reported MW (MEWS), executive skills (BRIEF-A), and functional impairment (WFRIS) separately as dependent variables. We hypothesized that MW frequency would be associated with all these clinical measures.

Analysis 2: To test our hypothesis for differences in the frequency of MW and in the context regulation of MW under increasing demand on working memory in the MWT, we tested the effects of condition (0-back vs. 1-back), group (ADHD vs. control) and group-by-condition interaction on MW frequency with repeated measures general linear models. We predicted a significant interaction whereby controls would show less frequent MW during the difficult (1-back) compared to the easy (0-back) condition (context regulation), whereas individuals with ADHD would mind wander to a similar degree during both conditions (deficient context regulation).

Similarly, to test our hypothesis of group differences in the overall frequency of MW and in context regulation of MW under increasing demand on sustained attention (SAT), the effects of condition (2, 5, 8 s), group (ADHD vs. controls) and group-by-condition interaction on MW frequency were examined with repeated measures general linear models. The frequent 1-s delays were not included in the analysis because there were no MW thought probes during these intervals. We expected a significant group by delay difficulty interaction, whereby controls would maintain continuous task focus/low MW frequency (context regulation) whereas individuals with ADHD would maintain high MW frequency across increasing delays (deficient context regulation). Further, we predicted an overall higher frequency of MW in ADHD individuals compared to controls during both tasks.

We also controlled for the effect of working memory capacity (difference in accuracy/incorrect responses between the 0-back and 1-back conditions in the MWT) on MW frequency (Analysis 2) because it has been proposed as a modulator of MW frequency (Kane & McVay, 2012; Mrazek et al., 2012).

Analysis 3: In analyses of cognitive performance (MRT, RTV, error rate), we tested the effect of condition (1-back vs. 0-back) in the MWT and the effect of delay (1 s vs. 2 s vs. 5 s vs. 8 s) in the SAT, group (ADHD vs. control) and group-by condition interaction with repeated measures general linear models for each task separately. To investigate the hypothesis that MW frequency explains measures of task performance statistically, we repeated these analyses using the probe-derived MW frequency during the MWT for the analysis of MWT performance and during the SAT for the analysis of SAT performance. After the end of each delay/interval, there was a stimulus and a response (a delay-affected trial). We calculated all SAT measures based on this trial (a stimulus and a response following straight after the duration of the delay) to study the effect of delay.

Given the large number of hypotheses tested in Analyses 1, 2, and 3, results were corrected for multiple testing using a false discovery rate (FDR) threshold based on the total number of comparisons in each task. FDR significant *p*-values were equal or lower than 0.032 for the MWT and equal or lower than 0.039 for the SAT.

Although there were no group differences for age between groups (Table 1), individuals with ADHD were marginally older than controls. We therefore covaried for age in Analysis 2 and 3 (Supplemental Analysis 1). The findings remained unchanged.

## Results

### Analysis 1: Associations Between Experimental MW Frequency and ADHD and MW Rating Scale Measures

MW frequency in both tasks was associated positively and strongly with all measures of ADHD symptoms, self-reported MW-D, executive skills and functional impairment (Table 2), but not with MW-D. All significant associations survived correction for multiple comparisons.

**Table 2.** Association of MW Frequency During Task Performance With MW, Clinical, and Functioning Measures.

	MW frequency during MWT			MW frequency during SAT		
	β	95% CIs	*p*	β	95% CIs	*p*
MEWS	0.67	0.45; 0.90	<.0001**	0.66	0.44; 0.88	<.0001**
MW-S	0.63	0.39; 0.88	<.0001**	0.67	0.44; 0.90	<.0001**
MW-D	0.20	−0.10; 0.49	.13	0.16	−0.14; 0.46	.29
DIVA inattention	0.74	0.52; 0.95	<.0001**	0.77	0.58; 0.97	<.0001**
DIVA hyperactivity-impulsivity	0.67	0.44; 0.91	<.0001**	0.66	0.44; 0.89	<.0001**
BRIEF	0.77	0.57; 0.97	<.0001**	0.73	0.54; 0.92	<.0001**
WFRISS	0.62	0.38; 0.87	<.0001**	0.49	0.25; 0.73	<.0001**

*Note.* MWT = Mind Wandering Task; SAT = Sustained Attention Task; MEWS = Mind Wandering Excessively Scale; MW-S = Spontaneous Mind Wandering (Seli et al., 2015); MW-D = Deliberate Mind Wandering (Seli et al., 2015); MW F = Mind Wandering frequency; MRT = mean reaction time; RTV = reaction time variability; BRIEF = Behavioral Rating Inventory of Executive function; WFRISS = Weiss Functional Impairment Rating Scale Self Report; DIVA = Diagnostic interview for ADHD in Adults.

* Significant at *p* ≤ .05, **significant at *p* ≤ .001, *d* ≥ 0.20 indicating a small effect size. OEs have been calculated by dividing the total number of errors by the number of trials.

### Analysis 2: Mind Wandering Frequency and Context Regulation of MW During the Mind Wandering Task

#### MW frequency

**Figure 3.**
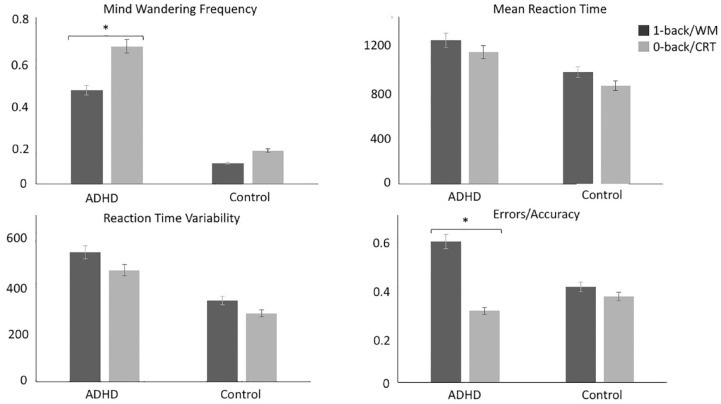
Comparisons between ADHD and control groups on MW, clinical and task performance measures during MWT. Individuals with ADHD reported decreased MW frequency during the 1-back (working memory) compared to the 0-back (choice reaction) condition contrasting no difference between conditions in controls (top left). Individuals with ADHD made slower (top right) and more variable (bottom left) responses compared to controls across both conditions. There were no group-differences for accuracy. However, individuals with ADHD made more incorrect responses during 1-back compared to the 0-back condition (bottom right). The mean in each condition for each group is shown. *p ≤ .05.

There was a significant effect of condition (*p* < .001), group (*p* < .001) and condition-by-group interaction (*p* = .026) (Figure 3). The main condition effect indicated that MW frequency was greater during the choice reaction condition (0-back) compared to the working memory (1-back) condition in both groups. Individuals with ADHD reported greater overall MW frequency compared to controls, as suggested by the main group effect (Table 3). However, the significant interaction indicated that the difference between the ADHD and control group in MW frequency was greater in the 0-back than in the 1-back condition. Post hoc analyses showed that individuals with ADHD showed more frequent MW during the 0-back compared to the 1-back (*p* = .001) (i.e., context regulation), whereas the difference between conditions in controls was not statistically significant (*p* = .090). After adding working memory capacity as a covariate, the main effect of condition (*p* = .001) and group (*p* < .001) as well as the condition-by-group interaction (*p* = .020) remained unchanged.

**Table 3.** Comparisons Between ADHD and Control Groups on MW, Clinical, Functioning, and Task Performance Measures.

Group comparisons		ADHD	Control	*d*	*p*
		Mean ± *SD*	Mean ± *SD*		
*MW scales*					
MEW-S		27.78 ± 7.19	5.31 ± 5.26	**3.56**	<.001***
MW-S		24.37 ± 3.47	12.58 ± 5.91	**2.43**	<.001***
MW-D		17.52 ± 7.51	15.69 ± 6.47	0.26	.330
*DIVA ADHD symptoms*					
Inattention		8.63 ± 0.63	0.56 ± 1.02	**9.50**	<.001***
Hyperactivity-impulsivity		7.06 ± 2.02	1.0 ± 0.88	**3.80**	<.001***
*Functioning scales*					
BRIEF		102.37 ± 20.21	23.07 ± 27.73	**3.27**	<.001***
WFRISS		83.81 ± 36.94	13.36 ± 10.46	**2.60**	<.001***
*Mind wandering memory task*					
MW frequency	1 back	0.45 ± 0.30	0.10 ± 0.12	**1.53**	<.001***
	0 back	0.66 ± .0.21	0.16 ± 0.19	**2.50**	<.001***
MRT	1 back	1,204.18 ± 292.25	938.71 ± 233.27	**1.00**	.001***
	0 back	1,105.05 ± 356.92	824.64 ± 215.73	**0.95**	.001***
RTV	1 back	544.41 ± 141.04	341.67 ± 139.83	**1.44**	<.001***
	0 back	468.38 ± 254.55	288.33 ± 172.56	**0.85**	.004*
Accuracy	1-back	0.59 ± 0.37	0.40 ± 0.35	*0.53*	.090
	0 back	0.30 ± 0.27	0.36 ± 0.32	0.20	.480
Working memory capacity		−0.29 ± 0.37	−0.06 ± 0.50	*0.52*	.048
*Sustained attention task*					
MW frequency	2 s	0.53 ± 0.32	0.13 ± 0.19	**1.52**	<.001***
	5 s	0.69 ± 0.31	0.13 ± 0.17	**2.24**	<.001***
	8 s	0.68 ± 0.34	0.18 ± 0.19	**1.82**	<.001***
MRT	1 s	315.14 ± 25.30	287.45 ± 31.33	**0.97**	.001***
	2 s	379.00 ± 29.18	370.48 ± 37.76	0.25	.381
	5 s	395.70 ± 25.71	378.01 ± 33.30	*0.59*	.050
	8 s	406.23 ± 27.69	379.49 ± 34.29	*0.52*	.003**
RTV	1 s	67.61 ± 9.84	55.45 ± 9.10	**1.28**	<.001***
	2 s	52.69 ± 11.31	49.25 ± 11.66	0.30	.291
	5 s	51.70 ± 8.27	50.54 ± 10.33	0.12	.660
	8 s	50.37 ± 10.93	51.20 ± 8.12	0.09	.762
OE	1 s	0.05 ± 0.04	0.02 ± 0.02	**0.95**	<.001***
	2 s	0.06 ± 0.04	0.04 ± 0.03	*0.57*	.050
	5 s	0.08 ± 0.05	0.05 ± 0.04	*0.66*	.010**
	8 s	0.09 ± 0.04	0.06 ± 0.04	*0.75*	.020**

*Note.* MEWS = Mind Wandering Excessively Scale; MW-S = Spontaneous Mind Wandering (Seli et al., 2015); MW-D = Deliberate Mind Wandering (Seli et al., 2015); MW F = Mind Wandering frequency; MRT = mean reaction time; RTV = reaction time variability; OE = omission errors; BRIEF = Behavioral Rating Inventory of Executive function; WFRISS = Weiss Functional Impairment Rating Scale Self Report; DIVA = Diagnostic interview for ADHD in Adults.

* Significant at *p* ≤ .032, **significant at *p* ≤ .039, ***significant at *p* ≤ .001, bold: *d* ≥ 0.80 indicating large effect size, italics: *d* ≥ 0.50 indicating a medium effect size, *d* ≥ 0.20 indicating a small effect size. OEs have been calculated by dividing the total number of errors by the number of trials.

### Mind Wandering Frequency and Context Regulation of MW During the Sustained Attention Task

#### MW frequency

**Figure 4.**
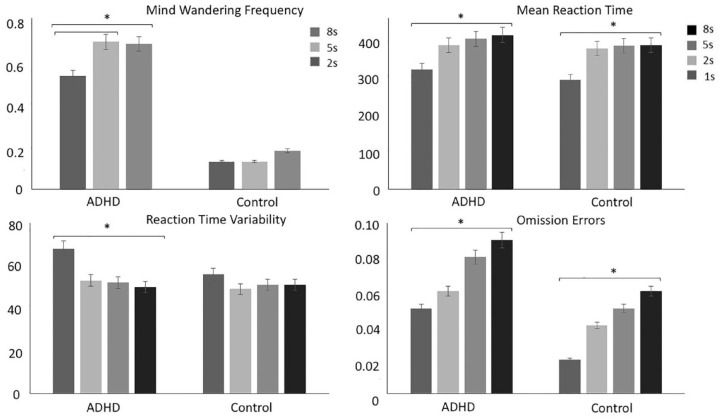
Comparisons between ADHD and control groups on MW, clinical and task performance measures during the SAT. Individuals with ADHD experienced greater MW frequency during 5 and 8 s delay compared to the 2 s delay contrasting no difference between conditions in controls (top left). Individuals with ADHD made slower responses compared to controls. Both controls and individuals ADHD reported slower responses with increasing delays (top right). Individuals with ADHD made more variable responses compared to controls. Individuals with ADHD made the most variable responses in the 1 s delays compared to the rest of the delays contrasting no difference between delays in controls. (bottom left). Individuals with ADHD made more omission errors compared to controls. Both groups made more omission errors with increasing delays (bottom right).

There was a significant main effect of delay (*p* = .004), group (*p* < .001) and a significant delay-by-group interaction (*p* = .020) (Figure 4). The main delay effect indicated that there was an increase in MW frequency with increasing delays. MW frequency was greater during 5 s (*p* = .007) and 8 s (*p* = .009) than during 2 s, while there was no difference between 8 and 5 s (*p* = .740). Individuals with ADHD reported mind wandering more frequently compared to controls during the task (Table 3). Post-hoc analyses following up the significant interaction effect showed that the difference between the ADHD and control groups was greater in the 5 and 8 s delay compared to the 2 s delay (Table 3). Individuals with ADHD reported more frequent MW during the 5 s (*p* = .020) and 8 s delay (*p* = .040) compared to the 2 s delay, but there was no difference between 5 and 8 s (*p* = .580) (i.e., deficient context regulation). In contrast, in controls MW frequency did not change significantly as function of increasing delays (2 s vs. 5 s *p* = .982, 2 s vs. 8 s *p* = .177, 5 s vs. 8 s *p* = .070). After adding working memory capacity as a covariate, the main effect of condition (*p* = .038) and group (*p* < .001) remained significant, while the delay-by-group interaction did not survive correction for multiple comparisons (*p* = .050).

### Analysis 3: Cognitive Performance and the Moderating Effect of MW Frequency

#### Mind wandering task

##### MRT: 0-back and 1-back

A significant effect of condition (*p* < .001) and group (*p* < .001) emerged, but there was no significant condition-by-group interaction (*p* = .951) (Figure 3). All participants were slower during the 1-back compared to the 0-back condition. Individuals with ADHD were overall slower compared to controls (Table 3). After adding MW frequency as a covariate, the condition effect remained significant (*p* = .013) and the interaction also remained non-significant (*p* = .820), while the group effect was no longer statistically significant (*p* = .080).

##### RTV: 0-back and 1-back

There was a main effect of condition (*p* = .009) and group (*p* < .001), but there was no significant condition-by-group interaction (*p* = .632) (Table 3). Responses were more variable in the 1-back compared to the 0-back condition. Individuals with ADHD had more variable responses compared to controls. After adding MW frequency as a covariate, the main effect of condition remained significant (*p* = .030), whereas the effect of greater RTV in the ADHD group was no longer statistically significant (*p* = .080).

#### Error rate

##### Incorrect responses/accuracy

There was a main effect of condition (*p* = .005) and a borderline significant condition-by-group interaction (*p* = .051), but no main group effect (*p* = .361). There were more incorrect responses in the 1-back compared to the 0-back condition. Unlike controls, who did not show differences between conditions (*p* = .777), individuals with ADHD made more incorrect responses during the 1-back compared to the 0-back (*p* = .001). This effect was no longer significant (*p* = .680) after adding MW frequency as a covariate.

#### Sustained Attention Task

##### MRT: Short frequent delay (1 s) and long infrequent delays (2, 5, 8 s)

There was a main effect of delay (*p* < .001) and group (*p* = .009), as well as a borderline significant condition-by-group interaction (*p* = .054) (Figure 4). Responses were fastest after the 1 s delays compared to the 2, 5, and 8 s delays (*p* < .001), after 2 s compared to the 5 s (*p* = .001) and 8 s (*p* < .001) delay, and after 5 s compared to 8 s delay (*p* = .030). Individuals with ADHD had slower responses across the entire duration of the task (Table 3). Controls made faster responses after 1 s delays compared to 2 s (*p* < .001), 5 s (*p* < .001), 8 s (*p* < .001), but response speed did not change after 2 s compared to 5 s (*p* = .080) and 8 s (*p* = .122), or 5 s compared to 8 s (*p* = .691). In contrast, individuals with ADHD made slower responses with increasing delays (1 s vs. 2 s, *p* < .001; 1 s vs. 5 s, *p* < .001; 1 s vs. 8 s, *p* < .001; 2 s vs. 5 s, *p* = .005; 2 s vs. 8 s, *p* < .001; 5 s vs. 8 s, *p* = .043). Both the effect of group (*p* = .480) and condition-by-group interaction (*p* = .451) were no longer significant after adding MW frequency as a covariate, while the effect of delay remained significant (*p* < .0001).

##### RTV: Short delay (1 s) and long delays (2, 5, 8 s)

There was a main effect of delay (*p* < .001), group (*p* = .039) and condition-by-group interaction (*p* = .001). Responses were more variable after 1 s delays compared to the 2, 5, and 8 s delays (*p* < .001). However, there was no difference in RTV after 2 s intervals compared to 5 s delays (*p* = .930), 2 s compared to 8 s delays (*p* = .921), 5 s compared to 8 s (*p* = .811). Compared to controls, individuals with ADHD had more variable responses during the 1 s intervals, but there was no difference between groups for 2, 5, and 8 s (Table 3). The group difference in RTV after 1 and 8 s delays was greater compared to 2 and 5 s, as indexed by the significant interaction. Controls did not show changes in RTV as a function of increasing delays (1 s vs. 2 s, *p* = .060; 1 s vs. 5 s, *p* = .060; 1 s vs. 8 s, *p* = .080; 2 s vs. 5 s, *p* = .630; 2 s vs. 8 s, *p* = .461; 5 s vs. 8 s, *p* = .671), whereas individuals with ADHD had more variable responses after 1 s delays compared to 2, 5, and 8 s delays (*p* < .001). The ADHD group also did not show modulation in RTV after 2 s compared to 5 s (*p* = .630) and 8 s (*p* = .450), or 5 s versus 8 s (*p* = .572). After adding MW frequency as a covariate, the effect of group (*p* = .100) and condition-by-group interaction (*p* = .171) were no longer significant, but the main effect of condition remained significant (*p* = .010).

#### Error rate: Short delay (1 s) and long delays (2, 5, 8 s)

##### Omission errors

There was a significant main effect of condition (*p* < .001), group (*p* = .004), but no significant condition-by-group interaction (*p* = .242). There were more OEs with increasing delays (2 s vs. 1 s (*p* < .001), 5 s vs. 1 s (*p* < .001), 8 s vs. 1 s (*p* < .001), 5 s vs. 2 s (*p* < .001), 8 s vs. 2 s (*p* < .001)), but there were no differences between 5 and 8 s delays (*p* = .08). Compared to controls, individuals with ADHD made more omission errors during all delays (Table 3). After adding MW frequency as a covariate, the main effect of group (*p* = .320) was no longer significant (*p* = .090), but the effect of condition (*p* = .010) and the interaction (*p* = .230) remained unchanged.

## Discussion

We first investigated the relationship between the experimental experience sampling measure of MW frequency and the clinical measures. We identified a strong association of the experimentally derived measures of MW frequency with ADHD symptoms, spontaneous MW, executive function, and functional impairment in daily life. This association confirmed the translational value of experimentally derived measures of MW frequency, as predictors of clinical outcomes, and as potential targets for treatment.

As expected, individuals with ADHD reported more frequent episodes of MW (50%–70%) compared to controls (10%–20%) across both tasks. The size of these effects was large (*d* = 1.5 to *d* = 2.5), providing clear evidence of increased MW frequency in ADHD compared to controls during both tasks (MWT and SAT). However, the frequency of MW in controls (10%–20%) was considerably lower than the frequency of MW (50%–55%) in population-based samples during the MWT (Konishi et al., 2015; Smallwood et al., 2013), suggesting differences between the population-based samples and our control sample. Our study selected controls for low levels of ADHD symptoms and no history of mental illness, whereas the previous population-based studies did not screen participants for symptoms of ADHD and thus might have included participants with a wider range of ADHD symptoms (Konishi et al., 2015; Smallwood et al., 2013).

The ADHD group experienced less MW under the high cognitive demand condition (working memory) compared to the low cognitive demand condition in the MWT, demonstrating context regulation of MW in response to higher demands on working memory, similar to that observed in population-based samples (Smallwood et al., 2013). In contrast, our control sample reported very low levels of MW frequency across both conditions. These findings appear contrary to our hypothesis of a deficit in context regulation of MW in ADHD, and intact context regulation of MW in controls. A potential explanation for the lack of context regulation in the control sample could be floor effects and sample choice.

Unlike the MWT, context regulation in the SAT reflects maintenance of increased task focus as demands on sustained attention increase. In that context, individuals with ADHD compared to controls experienced greater MW frequency with increasing inter-stimulus delays, indicating poor context regulation of MW in response to increasing demands on sustained attention. In contrast, controls maintained increased task focus with increasing inter-stimulus delays, suggesting effective adjustment to task demands (context regulation).

From these findings, we conclude that there is impaired context regulation of MW in response to demands on sustained attention, but not working memory. One possible explanation could be that processes underpinning sustained attention/vigilance might reflect a core deficit in ADHD, and/or potentially be more strongly related with MW. In contrast, working memory might reflect an additive impairment to ADHD, which does not play such a direct causal role in the maintenance of MW and ADHD symptoms. This view is supported by previous findings showing that sustained attention/vigilance measures show a stronger overlap in familial/genetic influences with ADHD than higher-level executive functions such as working memory (Kuntsi et al., 2010, 2014; Michelini, Kitsune, et al., 2018). Measures associated with sustained attention/vigilance also track the ADHD developmental course since they were found to be impaired in adolescents and adults with ADHD that persisted from childhood but not in remitted cases; whereas working memory and other measures of higher-level executive functions did not distinguish between ADHD persisters and remitters (Cheung et al., 2016; Michelini et al., 2016).

Consistent with these findings, a large adult outcome study found that the neural markers of attention processes and MW (atypical connectivity within the DMN, and between DMN and cortical control regions) (Christoff et al., 2009; Mason et al., 2007) were the strongest correlates of ADHD in adulthood, and differentiated ADHD persisters from remitters and non-ADHD controls (Sudre et al., 2017). These findings are also consistent with a meta-analysis of randomized controlled trials showing that working memory training results in improvement in working memory capacity but not in reduction in ADHD symptoms (Cortese et al., 2015). Furthermore, mindfulness-based interventions, thought to reduce ADHD symptoms (Cairncross & Miller, 2016), were also found to improve context regulation of MW which mediated an improvement in working memory capacity in a population-based sample (Mrazek et al., 2012), and regulation of DMN activity (Garrison et al., 2015).

Although working memory deficits are well-established in ADHD and viewed, in some models, as a core deficit leading to the inattentive symptoms of ADHD (Coghill et al., 2005; Kofler et al., 2014), alternative accounts (Sergeant, 2005) and evidence (Kim et al., 2014; Lenartowicz et al., 2019; Loo et al., 2007) suggest that deficits of sustained attention (i.e., encoding) may underlie/contribute to working memory deficits, at least in some individuals with ADHD. In a previous study, controls and individuals with ADHD with unimpaired working memory capacity showed context regulation of neural activity compared to individuals with ADHD with impaired working memory capacity (Mattfeld et al., 2016), supporting the alternative accounts and evidence.

Another key point is that increasing sustained attention and working memory demands are both associated with deficient context regulation of neural activity in individuals with ADHD compared to neurotypical individuals (Castellanos & Proal, 2012; Christakou et al., 2013; Cortese et al., 2012; Liddle et al., 2011; McLoughlin et al., 2014; Michelini et al., 2016, 2019; Rommel et al., 2016; Skirrow et al., 2015; van Rooij et al., 2015). This suggests that context regulation of underlying neural activity might still be compromised relative to controls.

In line with findings from population-based (Smallwood et al., 2013) and ADHD (Bozhilova et al., 2020; Christakou et al., 2013) samples, individuals with ADHD made slower and more variable responses. In particular, the ADHD group made more variable responses following more frequent 1 s delays compared to the longer delays, suggesting that the 1 s delays might appear easy and predictable and allow for more frequent MW. Individuals with ADHD also made more omission errors compared to controls during the SAT, supporting previous evidence of an attention-vigilance deficit as a core component of ADHD (Cheung et al., 2016; Michelini et al., 2016). The finding of more errors during the working memory than the choice-reaction condition in the ADHD group provides further evidence that working memory deficits are also common in individuals with ADHD, especially based on average estimates (Clark et al., 2007).

Based on our previous hypothesis that MW may explain underlying cognitive performance deficits of inattentive behaviors (Bozhilova et al., 2018), we tested whether experimental experience-sampling measures of MW frequency account for task performance impairments. In line with our hypothesis (Bozhilova et al., 2018), MW frequency explained statistically all cognitive performance differences between individuals with ADHD and controls, suggesting that MW could potentially underpin the cognitive performance deficits in ADHD.

## Implications for Neurocognitive Models of ADHD

Our findings have key implications for neurocognitive models of ADHD. The increased MW in response to demands on sustained attention in the ADHD group compared to controls suggests a core problem of allocating resources in response to increasing demands on sustained attention. This is in keeping with the cognitive-energetic (Sergeant, 2000; Sergeant et al., 2003) models of ADHD, which propose that the ability to preserve task performance under conditions of increasing attentional demand requires extra effort allocation. Congruently, once attentional demands increase, more frequent MW would be associated with depleted abilities to allocate cognitive resources, compromising task performance. Our finding of a lack of group differences in performance measures after controlling for MW frequency, supports these models and poor effort allocation in ADHD (Wiersema et al., 2006).

## Implications for Models of the Context Regulation of MW

Our findings may also have implications for the previous context regulation models: executive control failure (McVay & Kane, 2012), and executive control maintaining attentional resource (Smallwood, 2010). Based on previous studies using response inhibition tasks (Kane & McVay, 2012), we controlled for working memory capacity (using the difference in accuracy between the 0-back and 1-back conditions), to understand its potential effect on MW frequency. We found that working memory capacity did not account for either the overall increased MW frequency in ADHD during both tasks, or context regulation of MW under increasing demands on working memory in the MWT, and the lack of context regulation under increasing demands on sustained attention in the SAT. This finding does not appear to support either of the two previous models.

A potential explanation is that working memory capacity may moderate MW frequency and task performance only in tasks requiring restraint of habitual actions such as response inhibition. In line with this hypothesis, previously working memory capacity was found to predict MW frequency and task performance during a task probing response inhibition but not during a task probing vigilance and sustained attention (McVay & Kane, 2012) or even during tasks such as the MWT (Poole & Kane, 2009).

## Limitations and Future Directions

This study has three main limitations. First, the sample size is relatively small, and could only detect medium-to-large effects as significant. Second, we used differences in error rate only, as a proxy of working memory capacity, which limits our interpretation. Third, the tasks were not sufficiently difficult for controls. Future research should include a larger sample size, more difficult WM conditions such as 3 or 4-back conditions, or an additional measure of working memory capacity (i.e., the difference between digit span forward and backwards (Meule, 2017)) and an easier task condition (i.e., long, same-length, predictable intervals) and a harder task condition (2-back, 3-back) to elicit MW and task focus episodes in both groups. Future work should also include repeated measures design to enable causal modeling to investigate whether there is a context regulation at the neural level.

## Conclusion

Individuals with ADHD showed context regulation of MW frequency in response to increasing working memory load, but not in response to increasing sustained attention load. In contrast, controls maintained low levels of MW frequency during both tasks with no evidence of context regulation, presumably due to a floor effect. Working memory capacity did not account for these findings, which might be a task-dependent effect. Alternatively, a deficient context regulation of MW during increasing demands on sustained attention may reflect a core process in ADHD and give rise to other neurocognitive deficits. These findings suggest implications for neurocognitive models of ADHD/MW.

## Supplemental Material

Supplementary_material – Supplemental material for Context Regulation of Mind Wandering in ADHD Supplemental material, Supplementary_material for Context Regulation of Mind Wandering in ADHD by Natali Bozhilova, Giorgia Michelini, Christopher Jones, Jonna Kuntsi, Katya Rubia and Philip Asherson in Journal of Attention Disorders
