# Supplementary material for: Context Regulation of Mind Wandering in ADHD
Source: J Atten Disord. 2020 Sep 18;25(14):2014–27. doi: 10.1177/1087054720956714 (PMC8527550; doi:10.1177/1087054720956714)
Supplement: Supplementary_material – Supplemental material for Context Regulation of Mind Wandering in ADHD [file Supplementary_material.pdf]

Supplementary Material

Supplementary material 1: ADHD assessments

The Diagnostic Interview for ADHD in Adults (DIVA) (DIVA 2.0, Kooij, 2012), a semi-structured interview designed to evaluate DSM-IV criteria (DSM-5 criteria also applied) for ADHD, was conducted with all ADHD and control participants by trained interviewers. All interviews were reviewed by a psychiatrist specialising in adult ADHD (PA) to confirm diagnostic status**.**

Supplementary material 2: Self-reported measures

Spontaneous Mind Wandering

Spontaneous MW was measured using the Mind Excessively Wandering Scale (MEWS), a 12-item self-report measure reflecting descriptions of MW in ADHD. The 12-item MEWS captures the subjective experience of MW typical of individuals with ADHD, including thoughts constantly on the go, thoughts flitting from one topic to another, and multiple thoughts at the same time (Mowlem et al., 2016). The scale shows excellent internal consistency ($\alpha > .90$), as well as high sensitivity and specificity for ADHD compared to controls (both around 90%, area under the curve (AUC) =.97; Mowlem et al., 2016), and has been validated as a measure of spontaneous MW in a large population sample (Mowlem 2019). This scale measures the same construct/process across sex and diagnostic status (Mowlem et al., 2019). The total score was used to generate a self-report measure of MW.

The 4-item Mind Wandering - Deliberate (MW-D) scale and the 4-item Mind Wandering - Spontaneous (MW-S) scale (Carriere, Seli, & Smilek, 2013) were also used. These scales include items that measure controlled, deliberate MW (MW-D), such as *'I allow my mind to wander on purpose'* and uncontrolled, spontaneous MW (MW-S), such as '*I find my thoughts wander spontaneously*'. Both scales show high reliability (MW-S: $\alpha > 0.88$, MW-D: $\alpha > 0.89$) (Seli, Carriere, & Smilek, 2015). The MEWS correlates strongly with the MW-S scale ($r=.76$, $p<0.0001$), but not with MW-D ($r=0.05$, $p=0.06$), indicating that MW-S (not MW-D) is captured by the MEWS in individuals with and without ADHD (Mowlem et al., 2019). The total score for each subscale was used to provide an estimate of MW-S and MW-D.

Executive function skills

Self-reported executive function skills in daily life were investigated with a validated 30-item questionnaire, the Behavioural Rating Inventory of Executive function (BRIEF-A) (Roth et al., 2013). The total score was used to provide an index of executive functioning. The BRIEF-A has demonstrated moderate to high internal consistency (α =0.73 to 0.90) and high test-retest reliability (r=0.82 to 0.93) (Roth et al., 2015). This measure shows strong correlations with ADHD (Toplak, Bucciarelli, Jain, & Tannock, 2008).

Functional impairment

The Weiss Functional Impairment Rating Scale Self Report (WFIR-S) (Weiss et al., 2007) is a 59-item self-report measure which enquires about difficulties in a number of functional domains in the past month. The total score was used to provide an overall measure of functional impairment in daily life. The WFIR-S has demonstrated strong (α= 0.70 to 0.90) internal consistency and moderato to high test-retest reliability (r=0.50 to 0.70) (Weiss, McBride, Craig, & Jensen, 2018). The scale also shows strong correlations with ADHD (Thompson, Lloyd, Joseph, & Weiss, 2017).

Supplementary Analysis

Individuals with ADHD and controls were matched on age (Table 1). However, the lack of group differences was marginal (p=0.06). To test whether age has an effect on our primary results, we performed Analysis 2 and Analysis 3 by adding age as a covariate (Supplementary Analysis 1).

Supplementary Analysis 1: Controlling for the effect of age in Analysis 2 and Analysis 3

MWT

MW frequency

After adding age as a covariate, the main effect of group ($p<0.001$) and the condition-by-group interaction ($p=0.026$) remained significant, but the main effect of condition was no longer significant ($p=0.102$).

MRT

After adding age as a covariate, the main effect of group ($p=0.001$) remained significant, and the condition-by-group interaction ($p=0.702$) remained non-significant, but the main effect of condition was no longer significant ($p=0.807$).

RTV

After adding age as a covariate, the main effect of group ($p<0.001$) remained significant, and the condition-by-group interaction ($p=0.627$) remained non-significant, but the main effect of condition was no longer significant ($p=0.392$).

Error rate (accuracy)

After adding age as a covariate, the main effect of group ($p=0.216$) remained non-significant, and the condition-by-group interaction ($p=0.031$) remained significant but the main effect of condition was no longer significant ($p=0.068$).

SAT

MW frequency

After adding age as a covariate, the main effect of group ($p<0.001$) remained significant and the condition-by-group interaction ($p=0.050$) remained significant, but the main effect of condition ($p=0.423$) was no longer significant.

MWT

MRT

After adding age as a covariate, the main effect of condition ($p<0.0001$), group ($p=0.040$) and the condition-by-group interaction ($p=0.037$) remained significant.

RTV

After adding age as a covariate, the main effect of group ($p=0.002$) and the condition-by-group interaction ($p=0.004$) remained significant, but the main effect of condition was no longer significant ($p=0.367$).

Error rate (omission errors)

After adding age as a covariate, the main effect of group ($p=0.008$) remained significant and the condition-by-group interaction ($p=0.236$) remained non-significant, but the main effect of condition was no longer significant ($p=0.511$).

References:

Carriere, Jonathan SA, Paul Seli, and Daniel Smilek. 2013. "Wandering in Both Mind and Body: Individual Differences in Mind Wandering and Inattention Predict Fidgeting." *Canadian Journal of Experimental Psychology/Revue Canadienne de Psychologie Expérimentale* 67(1):19.

Kooij, JJ Sandra. 2012. *Adult ADHD: Diagnostic Assessment and Treatment*. Springer Science & Business Media.

Mowlem, Florence D., Caroline Skirrow, Peter Reid, Stefanos Maltezos, Simrit K. Nijjar, Andrew Merwood, Edward Barker, Ruth Cooper, Jonna Kuntsi, and Philip Asherson. 2016. "Validation of the mind excessively wandering scale and the relationship of mind wandering to impairment in adult ADHD. " *Journal of attention disorders* 23, no. 6: 624-634.

Mowlem, Florence D., Caroline Skirrow, Peter Reid, Stefanos Maltezos, Simrit K. Nijjar, Andrew Merwood, Edward Barker, Ruth Cooper, Jonna Kuntsi, and Philip Asherson. 2019. "Validation of the Mind Excessively Wandering Scale and the Relationship of Mind Wandering to Impairment in Adult ADHD." *Journal of Attention Disorders* 23(6):624–34.

Roth, Robert M., Peter K. Isquith, Gerard A. Gioia, A. Roy, J. Besnard, C. Lancelot, and D. Le Gall. 2013. "BRIEF-A." *Human Development* 89(11):865–74.

Seli, Paul, Jonathan Smallwood, James Allan Cheyne, and Daniel Smilek. 2015. "On the Relation of Mind Wandering and ADHD Symptomatology." *Psychonomic Bulletin & Review* 22(3):629–36.

Thompson, Trevor, Andrew Lloyd, Alain Joseph, and Margaret Weiss. 2017. "The Weiss Functional Impairment Rating Scale-Parent Form for assessing ADHD: evaluating diagnostic accuracy and determining optimal thresholds using ROC analysis." *Quality*

of Life Research 26, no. 7: 1879-1885.

Toplak, Maggie E., Stefania M. Bucciarelli, Umesh Jain, and Rosemary Tannock. 2008. "Executive functions: performance-based measures and the behavior rating inventory of executive function (BRIEF) in adolescents with attention deficit/hyperactivity disorder (ADHD)." *Child Neuropsychology*15, no. 1: 53-72.

Weiss, M. D., B. L. Brooks, G. L. Iverson, B. Lee, R. Dickson, and M. Wasdell. 2007. “Reliability and Validity of the Weiss Functional Impairment Rating Scale.” Pp. 23–28 in *AACAP 54th Annual Meeting, Boston, MA*.
